# The garden asparagus (*Asparagus officinalis* L.) mitochondrial genome revealed rich sequence variation throughout whole sequencing data

**DOI:** 10.3389/fpls.2023.1140043

**Published:** 2023-03-27

**Authors:** Wentao Sheng, Jianlan Deng, Chao Wang, Quan Kuang

**Affiliations:** ^1^ Department of Biological Technology, Nanchang Normal University, Nanchang, Jiangxi, China; ^2^ School of Foreign Language, Nanchang Normal University, Nanchang, Jiangxi, China

**Keywords:** asparagus officinalis, mitochondrial genome, PacBio and Illumina sequencing, phylogenetic analysis, asparagus

## Abstract

Garden asparagus (*Asparagus officinalis* L.) is a horticultural crop with high nutritional and medical value, considered an ideal plant for sex determination research among many dioecious plants, whose genomic information can support genetic analysis and breeding programs. In this research, the entire mitochondrial genome of *A. officinalis* was sequenced, annotated and assembled using a mixed Illumina and PacBio data. The garden asparagus circular mitochondrial genome measures 492,062 bp with a GC value of 45.9%. Thirty-six protein-coding genes, 17 tRNA and 6 rRNA genes were annotated, among which 8 protein-coding genes contained 16 introns. In addition, 254 SSRs with 10 complete tandem repeats and 293 non-tandem repeats were identified. It was found that the codons of edited sites located in the amino acids showed a leucine-formation trend, and RNA editing sites mainly caused the mutual transformation of amino acids with the same properties. Furthermore, 72 sequence fragments accounting for 20,240 bp, presentating 4.11% of the whole mitochondrial genome, were observed to migrate from chloroplast to mitochondrial genome of *A. officinalis*. The phylogenetic analysis showed that the closest genetic relationship between *A. officinalis* with onion (*Allium cepa*) inside the *Liliaceae* family. Our results demonstrated that high percentage of protein-coding genes had evolutionary conservative properties, with Ka/Ks values less than 1. Therefore, this study provides a high-quality garden asparagus mitochondrial genome, useful to promote better understanding of gene exchange between organelle genomes.

## Introduction

The genus *Asparagus* includes several important plant resources used in traditional Chinese medicine. Garden asparagus (*Asparagus officinalis* L.) is the most traditionally significant and best-known plant in Asparagaceae owing to its economical, nutritional, and medical value (Norup et al., 2015; Zhang et al., 2022; García et al., 2023). Over the past 20 years, the acreage of this plant has expanded significantly worldwide, especially in China (Nothnagel et al., 2022). At present, the main commercial asparagus varieties are diploid varieties that originate from the original “Violet Dutch.” (Knaflewski, 1996). It has been demonstrated that asparagus germplasm resources have a narrow genetic base (Nothnagel et al., 2012; Amian et al., 2018; Mousavizadeh et al., 2021; Ranjbar et al., 2022). Therefore, expanding the genetic diversity is a prominent goal in asparagus breeding. Interspecies hybridization is an important method for enriching genetic diversity and cultivating new cultivars (Takeuchi et al., 2018; Moreno-Pinel et al., 2021; García et al., 2022; Nothnagel et al., 2022). However, at present, the genetic relationship among many plants in the genus *Asparagus* remains unknown, which has hindered advances in the genetics and breeding of *A. officinalis* (Kubota et al., 2012; Mousavizadeh et al., 2021; García et al., 2022). Genetic sequence information is useful for clarifying the phylogenetic relationships within this genus (Kubota et al., 2012; Castro et al., 2013; Mousavizadeh et al., 2021). Although molecular markers and genomic information derived from the nuclear and chloroplast genome, which can be used to analyze the phylogeny among asparagus resources, have been examined, the taxonomic relationships and evolutionary history of this genus remains poorly understood (Castro et al., 2013; Norup et al., 2015; Wong et al., 2022). Notably, research on the phylogenetic analysis of the mitochondrial genome of garden asparagus is lacking.

As important organelles, plant mitochondria are related to the energy metabolism of cells, which affects plant growth, development, reproduction, and immunity (Bergthorsson et al., 2005; Cheng et al., 2021; Sibbald et al., 2021). Margulis’s endosymbiosis theory states that mitochondria originate from archaea, which live in nucleated cells and integrate into their organelles (Knoop, 2004; Bock, 2017; Christensen, 2021). For most higher plants, the nuclear genome is parentally inherited, whereas chloroplast and mitochondrial genomes are inherited maternally (Bergthorsson et al., 2005; Fields et al., 2022). This genetic approach eliminates the influence of paternal genes, reduces the difficulty of genetic research, and is conducive to research on genetic mechanisms (Kozik et al., 2019; Small et al., 2020). In many higher plants, mitochondrial DNA (mtDNA) is regarded as a closed-circular double-stranded structure (Wu et al., 2022). In general, mtDNA usually consists of 32-67 genes with variable lengths of coding and non-coding regions (Møller et al., 2021). Compared with nuclear and chloroplast genomes, the plant mitochondrial genome is characterized by a larger genome, rearrangement, rich repeat sequence recombination, and frequent gene acquisition (via deletion, transfer, and duplication) (Bock, 2017; Wu et al., 2022). Furthermore, mtDNA contains comprehensive information, which is valuable for phylogenetic analysis. Therefore, mtDNA can be considered a useful resource for genetic research (Rice et al., 2013; Xue et al., 2022). At present, 10,123 complete chloroplast and 585 plant mitochondrial genomes are available on NCBI (https://www.ncbi.nlm.nih.gov/genome/browse#!/overview/) (as of February 10, 2023), which provides a basis for the study of mitochondrial genomics.

*A. officinalis* is a model horticultural plant that is used for several studies, including those on sex-determining mechanisms, chromosome evolution, and gender diversity (Harkess et al., 2017 & Moreno et al., 2018; Harkess et al., 2020; You et al., 2022). To date, the complete nuclear and chloroplast genomes have been reported in *A. officinalis* and registered in the NCBI database (https://ncbi.nlm.nih.gov/) (Harkess et al., 2017; Sheng et al., 2017). However, the only registered mitochondrial genome of garden asparagus currently appears in the form of a briefing, and a detailed mitochondrial genome structural analysis has yet to be performed in *A. officinalis* (Sheng, 2020). In this study, we aimed to: (1) comprehensively analyze the mitochondrial genome structure and sequence variations of garden asparagus; (2) assess the gene transfer between chloroplast and mitochondria of garden asparagus; (3) explore the phylogenetic relationships and perform comparative genomic analysis of garden asparagus.

## Materials and methods

### Mitochondrial genome sequencing

The plant material of the cultivar ‘Atlas’ in *A. officinalis* was first obtained from the greenhouse of Nanchang Normal University and was discriminated by Professor Guangyu Chen (Jiangxi Academy of Agricultural Sciences, Nanchang, China, http://www.jxaas.cn/index.html). The sample was deposited in the Botanical Specimen Museum (http://swx.ncnu.edu.cn), and its voucher specimen number was NCNU-B-1021. Then, young adventitious roots were sampled, and whole genomic DNA was extracted from the harvested roots using the modified CTAB method (Arseneau et al., 2017). Subsequently, Nanodrop, 1% agarose gel electrophoresis and Qubit3.0 were used for DNA purity, concentration, and integrity inspection, respectively. Qualified DNA samples were sent to BIOZERON Gene Technology (http://www.Biozeron.com/) for Illumina and PacBio sequencing.

### The mitochondrial genome assembly and annotation

We assembled PacBio’s long reads into contigs using the Hierarchical Genome Assembly Process workflow in SMRT Analysis software suite 2.3 (Pacific Biosciences Inc., CA, United States) (Xie et al., 2020). The Basic Local Alignment Search Tool (BLAST) was used to identify mitochondrial contigs in every draft assembly with reference to *Oryza sativa* (CP018169.1) and *Allium cepa* (NC_030100.1) (Chen et al., 2015). Then, a self-looping contig was chosen as the candidate mitochondrial genome, relying on the number of matched hits. Illumina short-read and PacBio long-read data were utilized for correcting hybrid error with racon (v1.4.20), pilon (v 1.23), (Walker et al., 2014; Vaser et al., 2016) and minimap2/miniasm (Li, 2016; Li, 2018). All raw reads (containing short-read and long-read data) were mapped into the assembled mitochondrial genome structure using Burrows-Wheeler Aligner and SAMtools (v0.1.19) (Li and Durbin, 2009; Li et al., 2009). This indicated that the correctness of this genome assembly with the Illumina short-read and PacBio long-read data was based on a near-even coverage. The mean depth of the assembled mitochondrial genome was 197× (long reads), indicating a high copy number in the garden asparagus.

### Repeat sequence analysis

The mitochondrial genome of *A. officinalis* was analyzed using the Geseq annotation website and was manually annotated (https://chlorobox.mpimp-golm.mpg.de/geseq.html).TRNAscan-SE software was used to predict the tRNA gene to determine its location using the default parameters (Schattner et al., 2005). The mitochondrial genome structure map was drawn using the organelle genome drawing software OGDRAW (http://ogdraw.mpimp-golm.mpg.de/cgi-bin/ogdraw.pl). The sequences most likely to be encoded by proteins were analyzed using the gene prediction software ORF Finder (https://www.ncbi.nlm.nih.gov/orffmder/).

The SSR repeats of the mitochondrial genome in *A. officinalis* were analyzed using MISA software (http://pgrc.ipkgatersleben.de/misa/) (Thiel et al., 2003). The length of tandem repeats and the minimum number of repeats were set as follows: repeat motifs (1, 2, 3, 4, 5, and 6 bases) with repeat numbers (8, 5, 4, 3, 3, and 3). The minimum distance between two SSRs was set to 100 bp. Tandem repeat detection was performed using tandem repeat finder software using the default parameter settings (Benson, 1999). To screen the type, length, and position of the scattered repeat sequences in *A. officinalis*, the REPuter software was used for prediction. The parameters were set as a minimum repeat sequence length of 30 bp, and the similarity between the repeat sequences was >90% (Kurtz et al., 2001).

### RNA editing site predication and Ka/Ks value analysis

The potential RNA editing sites of *A. officinalis* protein-coding genes were predicted using PREP-Mt software (http://prep.unl.edu/) with a cut-off value of 0.2 (Mower, 2009). We also calculated the values of synonymous (Ks) and non-synonymous (Ka) substitution rates among the protein-coding genes in the *A. officinalis* mitochondrial genome with six representative species (*Arabidopsis thaliana*, NC_037304, *Nicotiana tabacum*, NC_006581.1, *Triticum aestivum*, NC_036024.1, *Ginkgo biloba*, NC_027976.1, *Funaria hygrometrica*, KC784959.1, and *Chlorella sorokiniana*, NC_024626.1). MEGAX was used for sequence extraction and alignment (Kumar et al., 2018), and the Ka/Ks value was determined using DNAsP v.6.12.03 (http://www.ub.edu/dnasp/) (Rozas et al., 2017).

### DNA transfer between mitochondrial and chloroplast genome

It is common for DNA to migrate in plants, and its frequency varies among species. DNA migration often occurs during fertilization, gametogenesis, and autophagy (Sloan and Wu, 2014). We downloaded the chloroplast genome of *A. officinalis* (NC_034777.1) from the NCBI database (https://www.ncbi.nlm.nih.gov/nuccore/NC_034777.1). In this study, the mitochondrial genome contigs were compared with the whole chloroplast genome sequence of *A. officinalis* using Blastn to determine the migration gene sequence.

### Genomic comparison with other mitochondrial genomes

To determine the differences between the mitochondrial genomes of *A. officinalis* and the six species (*Arabidopsis thaliana*, *Nicotiana tabacum*, *Triticum aestivum*, *Ginkgo biloba*, *Funaria hygrometrica*, and *Chlorella sorokiniana*), the mitochondrial genome size, GC content, coding genes, gene sequence insertions, and deletions were statistically analyzed and compared in this study. The plant species used for comparative analysis included algae, bryophytes, ferns, gymnosperms, and angiosperms in key positions of mitochondrial phylogenetic evolution (Ye et al., 2017). The mitochondrial genome information was obtained from the NCBI organelle genome resource library (https://www.ncbi.nlm.nih.gov/genome/browse#!/overview/).

### Phylogenomic tree analysis

We downloaded the mitogenomes of *A. officinalis* and other 41 taxa from the NCBI database (http://www.ncbi.nlm.nih.gov/genome/organelle/) (**Supplementary Table S1**). Among these species, not only do they have complete mitochondrial genome sequences, but they are also positioned clearly in plant taxonomy and are widely applied in molecular systematic research (Bi et al., 2016; Ye et al., 2017).

Phylogenetic tree re-constructing was carried out on 23 conserved protein-coding genes (*nad1, nad2, nad3, nad4, nad4L, nad5, nad6, nad7, nad9*, *ccmB, ccmC, ccmFc, ccmFn, cox1, cox2, cox3, atp1, atp4, atp6, atp8, atp9, cob*, and *matR*), which were extracted by Perl scripts. We aligned these selected gene sequences with the Muscle function plug-in in MEGAX (Kumar et al., 2018) and deleted gaps and missing data manually. Finally, we chose the AIC standard of jModelTest2.1.5 software (http://evomics.org/learning/phylogenetics/jmodeltest/) to select the best alternative model for the processed nucleotide sequence as GTR+F+R5 and constructed a phylogenetic tree using the maximum likelihood method of PhyML3.0, according to the best model (http://www.atgc-montpellier.fr/phyml/?tdsourcetag=s_pcqq_aiomsg). The degree of support of the maximum likelihood method node was obtained through 1000 replications. The gymnospermous plant *Ginkgo biloba* and the bryophyta plant of *Marchantia paleacea* were selected as the outgroup.

## Results

### Mitochondrial genomic organization

**Figure 1:**
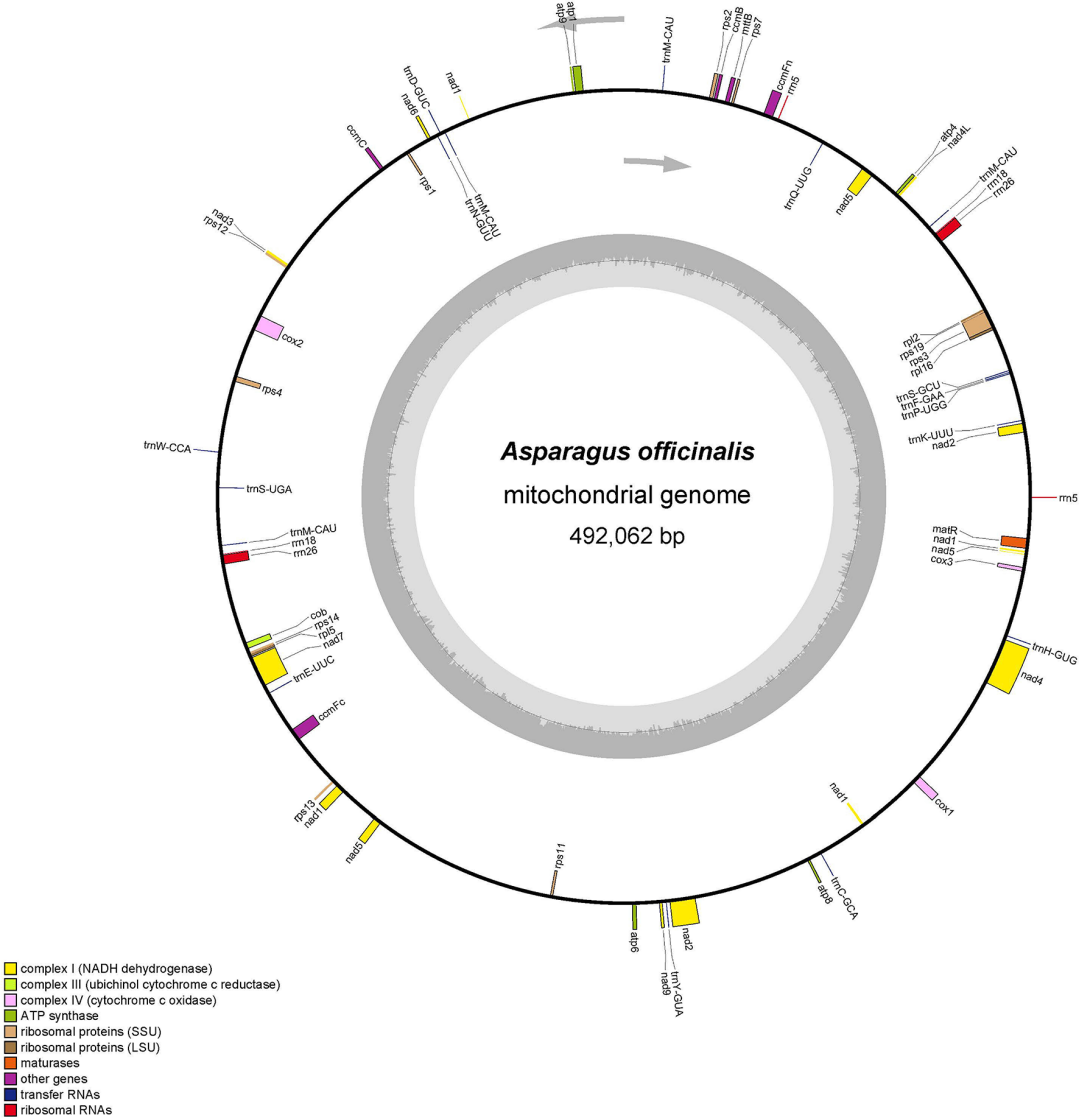
The mitochondrial genome organization structure of *A. officinalis*. The protein-coding region is marked with green, rRNA is marked with red, tRNA is marked with pink, and the repeat region is marked with brown in the circular mitochondrial genome structure. The copy starting point and direction of the L-chain replication are shown by a gray arrow.

The total mitochondrial genome length from garden asparagus was 492,062 bp, containing 58 genes (NCBI accession no. NC_053642.1), which displayed a typical single circular structure with 45.9% GC content (**Figure 1**). The base compositions of A, T, G, and C were 23.89, 24.35, 26.65, and 25.11%, respectively. Seventeen transfer RNA (tRNA), six ribosomal RNA (rRNA), and 36 protein-coding genes were annotated (**Table 1**). Based on their functional characteristics, the protein-coding genes were categorized into nine types: NADH dehydrogenase genes (9), ATP synthase genes (5), cytochrome c biogenesis genes (4), cytochrome c oxidase genes (3), ubiquinol cytochrome c reductase genes (1), transport membrane protein genes (1), maturase genes (1), large ribosomal proteins (LRP) genes (2), and small ribosomal proteins (SRP) genes (10) (**Table 1**). The whole length of the protein-coding genes was 31,773 bp, accounting for 6.46% of the whole mitochondrial genome. The intergenic region length was 460,289 bp, with 46.08% GC content, which comprised 93.54% of the mitochondrial genome size. Three rRNA genes were identified in the genome, including *rrn5* (117 bp), *rrn18* (1,970 bp), and *rrn26* (3,403 bp). Each rRNA gene contained two copies. In addition, 17 tRNA genes were identified in this genome, of which *trnM-CAU* had three copies and *trnM-CAU* was a chloroplast-derived gene.

**Table 1:** Gene organization in the mitochondrial genome of *A. officinalis*.

Types of genes	Gene name	Gene length	Start codon	Stop codon	Amino acid
NADH dehydrogenase	*nad1 (a)*	984	ACG	TAA	327
	*nad2 (a)*	1485	ATG	TAA	494
	*nad3*	357	ATG	TAA	118
	*nad4 (a)*	1488	ATG	TGA	495
	*nad4L*	324	CTG	TAA	107
	*nad5 (a)*	2013	ATG	TAA	670
	*nad6*	696	ATG	TGA	231
	*nad7 (a)*	1185	ATG	TAG	394
	*nad9*	573	ATG	TAA	190
**Cytochrome c biogenesis**	*ccmB*	621	ATG	TGA	206
	*ccmC*	747	ATG	TGA	248
	*ccmFc (a)*	1368	ATG	TAA	455
	*ccmFn*	1788	ATG	TAG	595
**Cytochrome c oxidase**	*cox1*	1584	ATG	TAA	527
	*cox2 (a)*	786	ATG	TAA	261
	*cox3*	798	ATG	TGA	265
**ATP synthase**	*atp1*	1530	ATG	TGA	509
	*atp4*	588	ATG	TAA	195
	*atp6*	813	ATG	TAA	270
	*atp8*	465	ATG	TAA	154
	*atp9*	231	ATG	TAA	76
**Maturases**	*matR*	1977	ATG	TAG	658
**Large ribosomal proteins (LRP)**	*rpl16*	558	ATG	TAA	185
	*rpl5*	567	ATG	TAA	188
**Small ribosomal proteins (SRP)**	*rps11*	501	ATG	TAA	166
	*rps12*	387	ATG	TGA	128
	*rps13*	351	ATG	TGA	116
	*rps14*	324	ATG	TAG	107
	*rps1*	501	ATG	TAA	166
	*rps19*	282	ATG	TAA	93
	*rps2*	696	ATG	TAG	231
	*rps3 (a)*	1677	ATG	TAG	558
	*rps4*	1035	ATG	TAA	344
	*rps7*	447	ATG	TAA	148
**Ubiquinol cytochrome c reductase**	*cob*	1182	ATG	TGA	393
**Transport membrane protein**	*mttB*	840	TTG	TAG	279
**Ribosomal RNAs**	*rRNA_18S (2)*	1970			
	*rRNA_26S (2)*	3403			
	*rRNA_5S (2)*	117			
**Transfer RNAs**	*trnC-GCA*	71			
	*trnD-GUC*	74			
	*trnE-UUC*	72			
	*trnF-GAA*	74			
	*trnH-GUG*	74			
	*trnK-UUU*	73			
	*trnM-CAU (b)*	73			
	*trnM-CAU (3)*	74			
	*trnN-GUU*	72			
	*trnP-UGG*	75			
	*trnQ-UUG*	72			
	*trnS-GCU*	88			
	*trnS-UGA*	87			
	*trnW-CCA*	74			

The numbers in brackets refer to the copy number of genes, lowercase a in brackets refers to the genes containing introns, and lowercase b in brackets refers to the genes from chloroplast genome.

For the annotated protein-coding genes, the ATG base combination was used as the initiation codon, except *nad1* (ACG), *nad4L* (CTG), and *mttB* (TTG). Furthermore, three types of termination codons (TAA, TGA, and TAG) were detected, with utilization frequencies of 55.6%, 25%, and 19.4%, respectively. And aundant intronic variation were shown in terrestrial plants. It was found that the number of introns in the mitochondrial genome is usually 1–4 (Li et al., 2021). The *rps3*, *ccmFc*, and *nad1* genes were annotated with one intron, *cox2* and *nad5* were composed of two introns, and *nad4*, *nad2*, and *nad7* had three introns each. Moreover, the length of the intron ranged from 861 bp (*nad7*) to 3,339 bp (*nad4*) (**Table 2**).

**Table 2:** The length of exon and intron in the *A. officinalis* mitochondrial genome.

Gene	Exon I (bp)	Intron I (bp)	Exon II (bp)	Intron II (bp)	Exon III (bp)	Intron III (bp)	Exon IV (bp)	Intron IV (bp)	Exon V (bp)
*rps3*	1,602	1,821	75						
*ccmFc*	609	955	759						
*cox2*	75	968	315	1,298	396				
*nad7*	144	861	69	2,836	246	1,535	261		
*nad4*	462	1,357	513	3,339	423	2,849	90		
*nad1*	387	NA	84	1,415	192	NA	63	NA	258
*nad2*	153	1,234	396	NA	153	2,581	594	1,393	189
*nad5*	225	868	1,221	NA	21	NA	396	935	150

### Repeat sequence identification

Based on the sequence length, repeat sequences can be divided into short, long, and tandem repeats (Alverson et al., 2011; Ye et al., 2017). Microsatellites or simple sequence repeats (SSRs) have a sequence repeat unit length of 1–6 base pairs. Microsatellites are characterized by abundant polymorphism, dominant inheritance, high occurrence frequency, uniform genome coverage, and speediness and simplicity in PCR detection (Liu et al., 2014). SSRs in the mitochondrial genome of *A. officinalis* were analyzed using tandem repeat analysis software (Benson, 1999). The result showed that 254 SSRs were detected in the *A. officinalis* mtDNA genome, of which the number of nucleotide repeats was monomer forms (206 SSRs), dimer forms (24), trimer forms (20), tetramer forms (28), and pentamer forms (3), respectively (**Supplementary Data Sheet 1**). The monomer SSR form had the highest proportion of repeat types, accounting for 81.1% of total SSRs. Among the monomer SSR, adenine (A) repeat type had the highest proportion (87.9%), AG repeat had the highest proportion (75%) of dinucleotide repeat types, and there was no sequence length with repeat numbers greater than 20 bp for all repeat types.

Tandem repeats, also known as satellite DNA, are characterized by repeat lengths of 1–200 bases, with varying numbers of repeats (Liu et al., 2014). They are mainly distributed in eukaryotic genome sequences. Ten perfect tandem repeats were identified with lengths ranging from 31 to 97 bp in the *A. officinalis* mitochondrial genome (**Table 3**). The majority of the ten tandem repeats were located in the intergenic region, but only one tandem repeat with a length of 48 bp was distributed within the *rpl16* gene.

**Table 3:** Characteristics distribution of perfect tandem repeats in *A. officinalis* mitochondrial genome.

No.	Size (bp)	Start	End	Repat sequence	Location
1	47	33,398	33,444	GGAATTGTCCGATCATAGCACGAT(x2.0)	*rpl16*
2	38	63,813	63,850	GTAGCTAGGAGTTGCTAGTT(x1.9)	NA
3	62	147,529	147,590	AAGCAGGGAAGGAAGCGCGAA(x2.9)	NA
4	38	248,301	248,338	CTAGCAACTCCTAGCTACAA(x1.9)	NA
5	35	327,069	327,103	TTCATATACAGCATTAT(x2.0)	NA
6	33	330,215	330,247	CGTCCTACGAATAC(x2.4)	NA
7	93	331,989	332,081	AGTTTGGATGTTCCAAGTACAAGTAGTAGT(x3.1)	NA
8	31	383,642	383,672	GAGTAGTTCCTCAA(x2.2)	NA
9	89	418,588	418,676	TTCACTCATGATCTGGCCTGACCTGGTCGACCCAATCATGATAT(x2.0)	NA
10	97	432,921	433,017	TAGGGAAGTCCGGGACAAAGTCCTCCTTCTTTGA(x2.8)	NA

**Figure 2:**
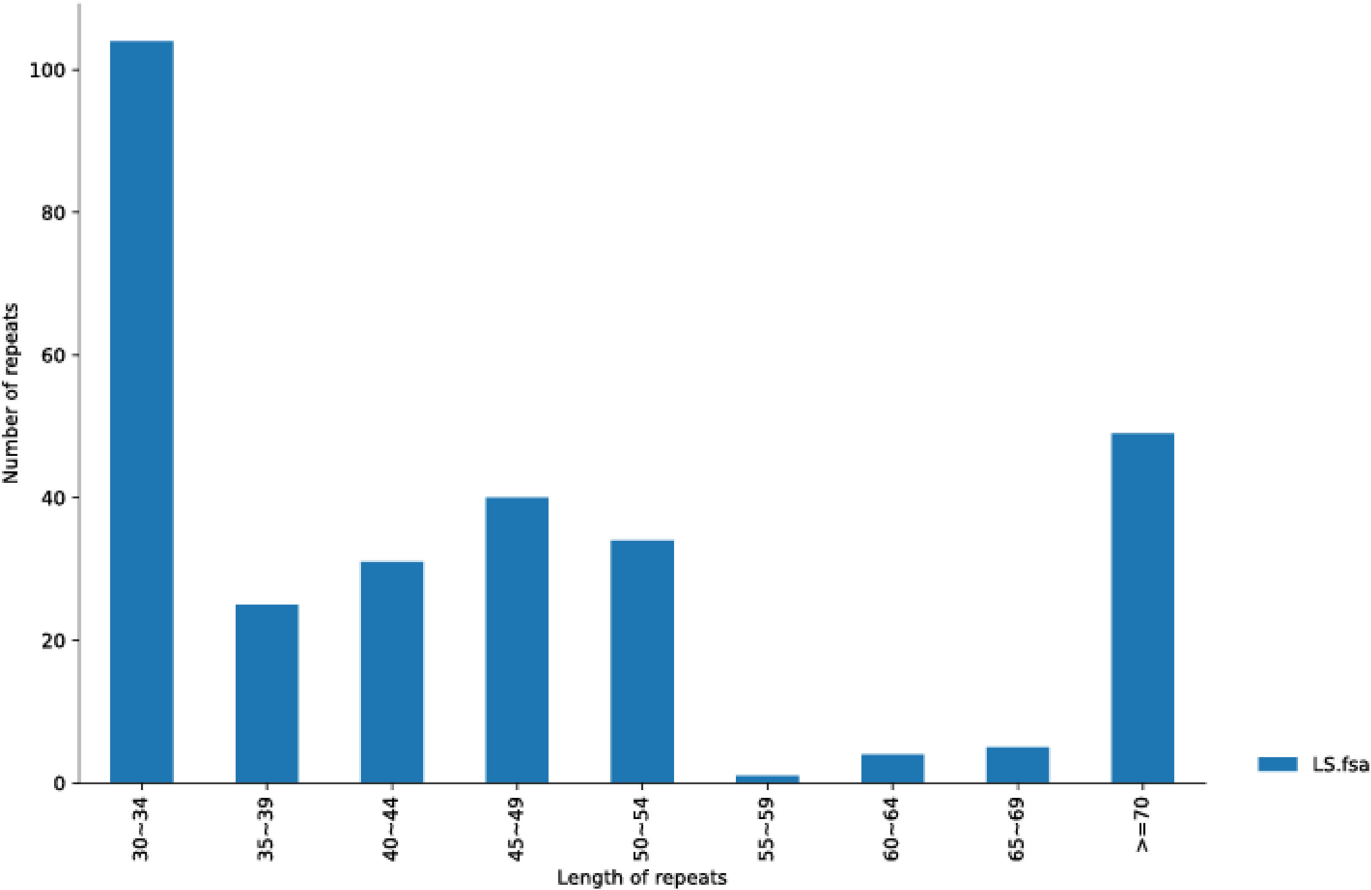
The repeat length distribution is shown in *A. officinalis* mitochondrial genome. The range size of the repetition length is displayed in the abscissa, and the number of repetitions is shown in the ordinate.

We also utilized Reputers software (http://bibiserv.techfak.uni-bielefeld.de/reputer/) to detect non-tandem repeat variations (Kurtz et al., 2001). For the long repeat sequence analysis, the parameters were set as minimum repeat size = 30 bp and edit distance = 3, to discriminate the following four repeat types: forward, reverse, complex, and palindromic repeat. It was found that 293 repeats had a length equal to or longer than 30 bp, of which 141 repeats were in the same direction, two were in the opposite direction, and 150 were in the palindrome structure (**Supplementary Data Sheet 2**). The longest forward repeat was 5,875 bp and the longest palindromic repeat was 12,348 bp. The length distribution of the entire repeat sequence is shown in **Figure 2**. The 30–34 bp segment was the most common, including 104 repeats. In contrast, the 55–59 bp segment was the least frequent type with only one repeat number.

### RNA editing sites prediction

**Figure 3:**
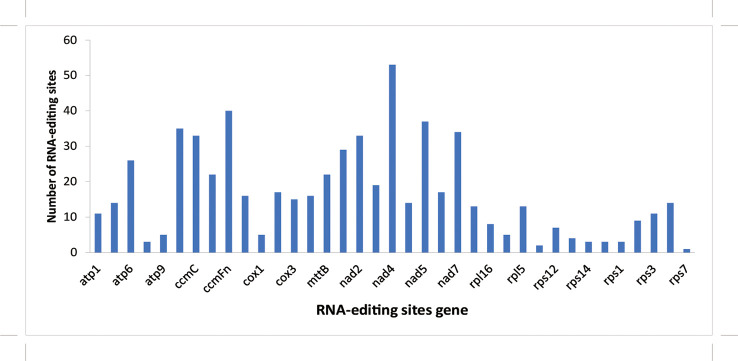
Prediction of RNA editing sites in protein-coding genes of mitochondrial genome in *A. officinalis*. The horizontal axis refers to the type of gene and the vertical axis refers to the number of editing sites.

In this study, the RNA editing site of each coding gene was analyzed using the prep software (http://prep.unl.edu/), and the change in the C-U editing site was predicted by homologous protein comparison (Bergthorsson et al., 2005; Mower, 2009). The results showed that *rps7* contained the least predicted editing sites (1), whereas *nad4* had the most predicted editing sites (53) among these protein-coding genes (**Figure 3**).

For all identified editing sites, 215 sites were checked in the first encoding position, accounting for 35.19%, while 595 sites were marked in the second encoding position, accounting for 64.81%. Furthermore, an interesting phenomenon occurred in the predicted sites, where two base sites (CCT to TTT, CCC to TTC) changed at the same time. Meanwhile, 32.68% (200) of the hydrophobic amino acid sites remained unchanged, 11.93% (73) of the hydrophilic amino acid sites did not change, 9.15% (56) of the total sites changed from hydrophobic to hydrophilic amino acids, 45.42% (278) of hydrophilic amino acids had turned into hydrophobic amino acids, and 0.82% (5) of amino acid coding sites resulted in stop codons, located in the *atp9* and *ccmFc* gene (CGA-TGA), *rps11* and *atp6* gene (CAA-TAA), and *rpl16* gene (CAG-TAG), respectively. In this study, the amino acids in the codons of the edited sites were found to exhibit a leucine formation trend, which was supported by the fact that 510 amino acid editing sites (83.4%) were predicted to be converted into leucine (**Table 4**).

**Table 4:** Characteristics of hydrophilic and hydrophobic changes in RNA editing sites.

Type	RNA-editing	Number	Percentage
hydrophobic	CCA (P)=>CTA (L)	50	32.68%
	CCG (P)=>CTG (L)	36	
	CCT (P)=>CTT (L)	32	
	CCT (P)=>TTT (F)	14	
	CCC (P)=>CTC (L)	11	
	CCC (P)=>TTC (F)	4	
	CTT (L)=>TTT (F)	30	
	CTC (L)=>TTC (F)	11	
	GCG (A)=>GTG (V)	5	
	GCT (A)=>GTT (V)	4	
	GCC (A)=>GTC (V)	2	
	GCA (A)=>GTA (V)	1	
hydrophilic	CGT (R)=>TGT (C)	32	11.93%
	CGC (R)=>TGC (C)	11	
	CAT (H)=>TAT (Y)	21	
	CAC (H)=>TAC (Y)	9	
hydrophobic-hydrophilic	CCT (P)=>TCT (S)	25	9.15%
	CCC (P)=>TCC (S)	15	
	CCA (P)=>TCA (S)	11	
	CCG (P)=>TCG (S)	5	
hydrophilic-hydrophobic	ACA (T)=>ATA (I)	5	45.42%
	ACG (T)=>ATG (M)	5	
	ACT (T)=>ATT (I)	4	
	ACC (T)=>ATC (I)	2	
	TCA (S)=>TTA (L)	76	
	TCT (S)=>TTT (F)	54	
	TCG (S)=>TTG (L)	50	
	TCC (S)=>TTC (F)	44	
	CGG (R)=>TGG (W)	38	
hydrophilic-stop	CGA (R)=>TGA (X)	2	0.82%
	CAA (Q)=>TAA (X)	2	
	CAG (Q)=>TAG (X)	1	

### Chloroplast-derived mitochondrial genome sequences

The plant mitochondrial genome is characterized by its sequence length size, genome structure and composition, gene content, and function (Kozik et al., 2019). In addition, fragment exchange between mitochondria and chloroplast is common. Approximately 5–10% of different species of the mitochondrial genome can find homologous sequences in its corresponding chloroplast genome (Sloan and Wu, 2014; Petersen et al., 2020). Homologous regions of the mitochondrial and chloroplast genomes were determined by Blastn (ncbi-blast-2.2.30+) comparison. As a result, 72 fragments of 20,240 bp were observed to migrate from the chloroplast to the mitochondrial genome of *A. officinalis*, accounting for 4.11% of the whole mitochondrial genome.

Moreover, four annotated gene fragments were transferred: including *atp1* (460 and 89 bp segment), *rrn26* (192, 192, 164, 164, 97, 97, 97, 97, 75, 69, and 69 bp segments), *trnM-CAU* (77 bp segment), and *trnF-GAA* (59 bp segment) (**Supplementary Table S2**). Among them, the transferred fragment in the *atp1* gene accounted for 35.9% of the total length, while the *rrn26* gene was 38.5%. However, nearly all *trnM-CAU* and *trnF-GAA* fragments were transferred. These data indicate that some rRNA genes, tRNA genes, and ATP synthesis-related genes are easy to transfer, presenting weaker sequence conservation during gene migration in *A. officinalis.*

### Phylogenetic inference

The phylogenetic tree rebuilt using conserved mitochondrial protein-coding genes can be helpful for determining the molecular evolutionary relationships of green plants (Duminil and Besnard, 2021; Xue et al., 2022). To ascertain the taxonomic status of the mitochondrial genome in *A. officinalis*, phylogenetic analysis was carried out with 41 other species, including 26 eudicotyledons (rosids, 19 species; asterids, 7 species), 13 monocotyledon plants, one gymnosperm plant, and one bryophyta plant (designated as the out-group). A phylogenetic tree was reconstructed after sequence comparison based on 23 conserved protein-coding genes from the selected species.

**Figure 4:**
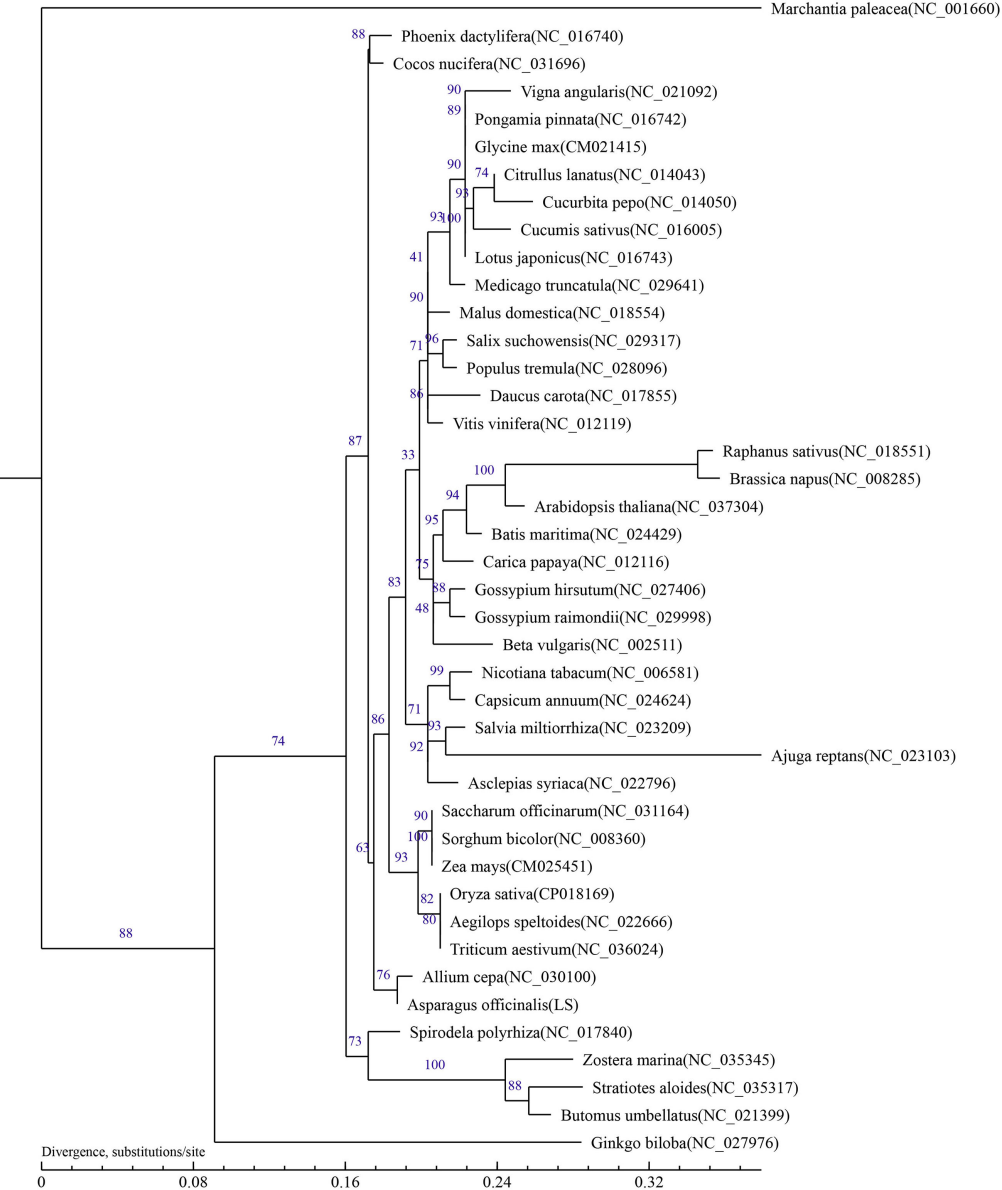
Phylogenetic tree inferred based on 23 conserved protein-coding genes of 42 typical plant mitochondrial genomes. Numbers on each node were bootstrap support values. *Marchantia paleacea* was used as the out-group.

The results showed that the phylogenetic trees strongly supported the partitioning of dicotyledons from monocotyledons and the separation of angiosperms from gymnosperms. In addition, taxa from 24 families (Leguminosae, Vitaceae, Rosaceae, Malvaceae, Caricaceae, Bataceae, Brassicaceae, Salicaceae, Cucurbitaceae, Apiaceae, Solanaceae, Amaranthaceae, Lamiaceae, Apocynaceae, Poaceae, Amaryllidaceae, Butomaceae, Arecaceae, Araceae, Hydrocharitaceae, Zosteraceae, Ginkgoaceae, and Marchantiaceae) were well clustered. The relationship in the phylogenetic tree was in line with the traditional taxonomic relationship of these species, indicating consistency between traditional classification and molecular classification at the family level. Phylogenetic analysis showed that *A. officinalis* had the closest genetic relationship with *Allium cepa* L.(**Figure 4**). To determine the phylogenetic relationship of *A. officinalis* in the genus *Asparagus*, it is necessary to obtain further mitochondrial genome information of smaller taxa.

### Comparative genomics

The genome size and GC content in organelles are the two principal features of mitochondiral DNA genome research (Ye et al., 2017; Robles and Quesada, 2021). Herein, we compared *A. officinalis* with the six typical plants (*Arabidopsis thaliana*, *Nicotiana tabacum*, *Triticum aestivum*, *Ginkgo biloba*, *Funaria hygrometrica*, and *Chlorella sorokiniana*) in terms of their mitochondrial genome characteristics. For the lower plant types, the proportion of protein-coding genes in *Chlorella sorokiniana* and *Funaria hygrometrica* was 42.26% and 29.51%, respectively, indicating that the coding sequences of genes accounted for a high proportion in the genome (**Table 5**). Among the higher plant types, the ratio of protein-coding genes in *A. officinalis* and *Arabidopsis thaliana* was 6.45% and 8.53%, respectively, showing that the coding sequence of genes occupies a lower proportion in the mitochondrial genome. Related to this, the number of non-coding sequences was roughly the same as that of coding gene sequences in lower plants, but accounted for a high proportion in higher plants. These data demonstrate that with the evolution of species, the number of non-coding sequences continues to increase, resulting in a significant increase in the mitochondrial genome, which plays an important role in the composition and structure of the genome. Moreover, rRNA and tRNA genes showed the same downward trend as the species evolved. The presence of a large number of cis-splicing introns in the mitochondrial genome is also an important reason for the change in mitochondrial genome size.

**Table 5:** Comparison of genome characteristics in seven plant mitochondrial genome.

Plants species	Family	Coding regions (%)				Non-coding regions (%)
		Protein-coding genes	Cis-spliced introns	rRNAs	tRNAs	
*Chlorella sorokiniana*	*Chlorellaceae*	42.26%	0.00%	7.03%	3.73%	46.98%
*Funaria hygrometrica*	*Funariaceae*	29.51%	29.15%	4.58%	1.65%	35.12%
*Ginkgo biloba*	*Ginkgoaceae*	9.95%	11.30%	1.44%	0.50%	76.80%
*Triticum aestivum*	*Poaceae*	7.39%	5.04%	3.00%	0.36%	84.20%
*Asparagus officinalis*	*Asparagaceae*	6.45%	5.24%	0.80%	0.26%	87.26%
*Nicotiana tabacum*	*Nicotianeae*	7.31%	5.71%	2.05%	0.40%	84.53%
*Arabidopsis thaliana*	*Brassicaceae*	8.53%	7.72%	1.42%	0.46%	81.87%

The GC content of our observed plant mitochondrial genome varied from 29.11% in *Chlorella sorokiniana* to 50.36% in *Ginkgo biloba*. The results showed that the genome size of angiosperms was higher than that of algae and gymnosperms, and its size also fluctuated significantly; however, its GC content fluctuated slightly and was conservative in angiosperm plants (**Supplementary Table S3**). For the mitochondrial protein-coding genes, we found that the mitochondrial complex I, III, IV, and V genes and the cytochrome C synthesis gene were not lost with strong conservatism. However, the ribosomal synthetic protein, tRNA, complex II, and *mttR* and *mattB* genes were often missing in different species with high volatility and weak conservatism during plant evolution. Meanwhile, some genes, such as *rps19*, became pseudogenes in *Triticum aestivum* (**Supplementary Table S3**).

### Substitution rate of protein-coding genes

Selection pressures containing the non-synonymous (Ka) and synonymous (Ks) nucleotide substitution models are useful parameters in molecular evolution research (Zhang et al., 2006; Shtolz and Mishmar, 2019). The Ka/Ks ratio can be used to justify the selective pressure on specific protein-coding genes in the process of evolution, among which Ka/Ks > 1 represents positive selection, Ka/Ks = 1 represents neutral selection, and Ka/Ks < 1 refers to negative selection (Li et al., 2009; Sloan et al., 2012).

**Figure 5:**
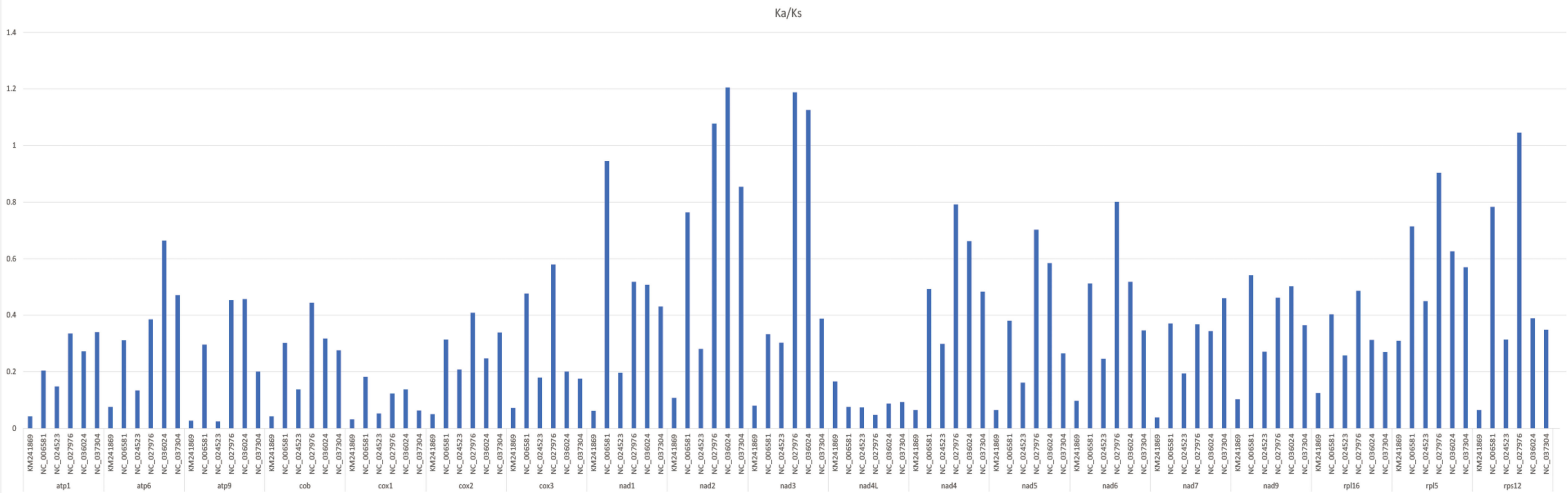
Ka/Ks of 18 similar protein-coding genes of *A. officinalis* versus six species. Deep gray and light gray boxes indicate Ka/Ks ratio of *A. officinalis* versus *Chlorella sorokiniana, Nicotiana tabacum*, *Funaria hygrometrica*, *Ginkgo biloba*, *Triticum aestivum, Arabidopsis thaliana*, respectively.

The Ka/Ks value of the common protein-coding genes was computed in the *A. officinalis* mitochondrial genome and compared to six typical plant species (*Arabidopsis thaliana*, *Nicotiana tabacum*, *Triticum aestivum*, *Ginkgo biloba*, *Funaria hygrometrica*, and *Chlorella sorokiniana*) (**Figure 5**). In total, the mean Ka/Ks value of 18 identical protein-coding genes was 0.35 in the seven analyzed species. The data showed that the Ka/Ks ratio of *A. officinalis nad2* and *nad3* compared to *Ginkgo biloba* and *Triticum aestivum* were larger than 1, and its ratio of *A. officinalis rps12* compared to *Ginkgo biloba* was higher than 1, indicating a positive selection effect on its evolution. However, the majority of the protein-coding genes with Ka/Ks values < 1 indicated a negative selection effect occurring in the genes of *A. officinalis* compared to the other six species. This indicates that most protein-coding genes in the mitochondrial genome of *A. officinalis* are highly conserved during the process of molecular evolution.

## Discussion

Generally, the plant mitochondrial genome is characterized by a complex composition, diverse structure, fluctuating non-coding sequences, and high recombination of repetitive sequences (Skippington et al., 2015; Small et al., 2020). Owing to these features, it is difficult to study plant mitochondria. In the current study, a strategy was proposed to obtain the plant mitochondrial genome based on the combined splicing of second- and third-generation whole-genome sequencing data. This principle benefits from the fact that the copy number of the plant organelle genome is much higher than that of its corresponding nuclear genome (Shtolz and Mishmar, 2019). Therefore, we applied a combination of Illumina and PacBio sequencing technologies to the study of plant mitochondria and obtained the mitochondrial genome sequence of *A. officinalis*, which is the first reported mitochondrial genome sequence in Asparagaceae and provides a reference for future mitochondrial genome research.

Herein, the mitochondrial genome of *A. officinalis* was assembled into a single circular molecule after genome sequencing, assembly, and gap-filling steps. Previous studies have found that the genome length of most plant mitochondria ranges from 200 to 700 kb, among which the smallest genome is *Viscum scurruloideum* (with only 66 kb) (Skippington et al., 2015) and the largest genome is *Silene conica* (11.3 Mb) (Richardson et al., 2013). At the same time, the content of genes also changed significantly, mainly in the range of 32 to 67 genes, with some genes, such as NADH dehydrogenase, losing or regaining their functions during evolution (Knoop, 2004). In this study, the mitochondrial genome of *A. officinalis* was assembled into a circular structure with a total length of 492,062 bp, encoding 58 genes, and the genome size was in the middle range. The gene types and numbers in *A. officinalis* mitochondria were consistent with the majority of reported circular mitochondrial genomes in most land plants (Alverson et al., 2010; Møller et al., 2021). Comparative genomic analysis revealed that the size of the genome contained in different plants was mainly caused by the spacer of the genes. A large number of unknown sequences and repetitive fragments are known to accumulate in the interval of genes. Furthermore, the intracellular transfer of genes can cause changes in the spacer (Choi et al., 2019; Fields et al., 2022). Accordingly, a large number of non-coding gene sequences were also found in the mitochondrial genome of *A. officinalis*, and abundant sequences from chloroplast genome transfers were observed.

Gene transfer between the chloroplast and mitochondrial genomes is an important feature of plant mitochondrial genome evolution (Notsu et al., 2002; Choi et al., 2019). Thus, tracking gene migration is essential to explore the evolution of the plant mitochondrial genome. This phenomenon is the main reason for the differences in the number of coding genes observed in different plant species (Sibbald et al., 2021). For example, the mitochondrial genome in *Amborella trichopoda* contains more than 250 coding genes, which may have been caused by horizontal gene transfer during evolution (Rice et al., 2013). The chloroplast genes in angiosperms are transferred to the mitochondrial genome (Sloan and Wu, 2014). Based on the data, it was found that 34 migration fragments were equal to or greater than l00 bp, of which the longest fragment was 2,013 bp. The transfer length of *rrn26* was 1,388 bp, whereas in *Suaeda gluaca*, the gene length was only 1,369 bp, which may be the reason for the large *rrn26* gene in *A. officinalis*.

The mechanism of male sterility in crops is related to RNA editing (Tang and Luo, 2018; Robles and Quesada, 2021). RNA editing is a means of modifying genetic information at the transcriptional level by inserting, knocking out, and replacing RNA base sequences. RNA editing in plants mainly occurs from cytosine (C) to uracil (U) (Malek et al., 1996; Tang and Luo, 2018). RNA editing regulates plant growth and development by correcting “wrong” sequences based on organelle transcripts. RNA editing usually occurs at the first or second base of the codon (Sloan and Wu, 2014; Christensen, 2021). By generating a new start codon, a new stop codon, or changing the sequence of amino acids, it promotes the formation of correct secondary protein structures, which plays important roles in gene regulation, ensuring the accurate expression of genetic information in cells (Small et al., 2020). In *A. officinalis*, the G/C codon usage is preferred. This preference may be due to the high binding energy of G/C codons, which is conducive to ensuring translation accuracy (Tang and Luo, 2018; Christensen, 2021). The tRNA genes encoded by the mitochondrial genome are highly conserved and can be divided into two categories: 10–12 tRNAs in mitochondria and 4–13 chloroplast-like tRNAs with high homology with chloroplast DNA in *A. officinalis*. RNA editing is ubiquitous in higher plant mitochondria and is an essential process for the production of functional proteins by mitochondria (Warren and Sloan, 2020). The start codon in the *A. officinalis* mitochondrial genome was different from the standard AUG start codon. Therefore, the discovery of RNA editing reasonably explains why mitochondria are inconsistent with the use of standard codons in nuclear heredity. Thus, in future research, sterile male line materials could be created according to the RNA editing sites of *A. officinalis* to promote genetic breeding.

Repetitive sequences play an important role in genome recombination, mediating frequent recombination and producing a large number of chimeric genes, leading to crop abortion (Wu et al., 2022). Differences in mitochondrial genome size in the same species are mainly caused by repetitive sequences, especially the noncoding sequences of gene spacers (Duminil and Besnard, 2021; Li et al., 2021). Recombination is the main mechanism underlying mitochondrial genome evolution (Tomal et al., 2019; Welchen et al., 2021). The difference between the mitochondrial genome size of dicotyledonous plants *Silene conica* (11,318,806 nt) and that of *Silene latifolia* (253,413 nt) in the Dianthus family were found to be approximately 44.7-fold (Sloan et al., 2012). The mitochondrial genome sizes of *Cucurbitaceae melon* (approximately 2.9 M) and watermelon (379,236 nt) differed by over seven-fold (Alverson et al., 2010). Altogether, 254 SSRs and 293 non-tandem repeats were identified in *A. officinalis* repeat types. Moreover, a large palindromic sequence (12,348 bp) was identified. Similar large fragments were detected in *Gossypium raimondii* (Bi et al., 2016), *Salix hypochonsis* (Ye et al., 2017), and *Acer truncatum* (Ma et al., 2022). This suggests that the mitochondrial genome is relatively complex, with the characteristics of structural polymorphism, composition heterogeneity, and gene sequence variability in *A. officinalis*.

Plant mitochondrial genomes are characterized by the usual structural rearrangement, the loss or acquisition of a large number of genes, the usual intragenic transfer and exogenous DNA transfer, highly variable RNA editing processes, and extremely low nucleotide mutation rates (Duminil and Besnard, 2021; Xue et al., 2022). This provides unique information for the study of systematic molecular evolution (Christensen, 2021; Fields et al., 2022). The analysis of plant mitochondrial gene homology can reflect the relationships and evolutionary history of different species (Kozik et al., 2019). Herein, the phylogenetic relationship in *A. officinalis* was rebuilt using mitochondrial genome information based on representative species at the family level. The results indicated that a clear taxonomic relationship was reflected with the traditional classification of taxa. To classify the phylogenetic relationship of the whole species of the genus *Asparagus*, more species in different subgenera were sequenced to straighten their development relationships and perform more effective interspecific hybridization breeding.

The GC content in the mitochondrial genome was compared to that of some selective species and was found to be highly conserved in higher plants. Genome comparison and Ka/Ks analysis of protein-coding genes provide a good theoretical basis for exploring mitochondrial genome evolution. Generally, few coding genes in this study were affected by the positive selection effect, which is similar to existing research reports (Cheng et al., 2021; Ma et al., 2022). At the same time, this study also identified genes subject to neutral and negative selection pressure, most of which were affected by the negative selection effect. This analysis further indicated that protein-coding genes in the mitochondrial genome were conserved in green plants.

Previous studies have found that cytoplasmic male sterility is associated with changes in mitochondrial genes (Bi et al., 2016; Robles and Quesada, 2021). Therefore, the study of the mitochondrial genome may provide insights into the genetics and breeding of this plant. The expounding of gene composition, structure, and function in the mitochondrial genome is the basis of fertility, molecular-assisted breeding, and genetic evolution in plants (Shtolz and Mishmar, 2019; Ma et al., 2022). Given that garden asparagus is an important crop, its economic, edible, and nutritional health care value has been verified. As the most important plant of the family *Asparagiaceae*, garden asparagus is characterized by sex diversity, diverse propagation methods (e.g., ramet propagation, seed, and cutting propagation), a small chromosome genome (2n = 20), self-cross or hetero-cross pollination, and mature genetic transformation systems (Moreno et al., 2018; Moreno-Pinel et al., 2021; Encina and Regalado, 2022; García et al., 2023). Therefore, garden asparagus is an ideal material for many studies of dioecious plants. The garden asparagus industry has proposed it as a model plant for sex-determining mechanisms (Takeuchi et al., 2018; Harkess et al., 2020; Ahmad et al., 2022; You et al., 2022). Moreover, abundant variations were detected in garden asparagus mitochondria, including the accumulation of repetitive sequences, the acquisition of unknown sequences, variations in intron size, and variations in the number and size of genes. The mitochondrial gene composition and size, repeat sequences, and chloroplast-derived sequences provide an important basis for studying the origin and evolution of garden asparagus. Phylogenetic analysis using new genome sequences will provide insights into evolutionary pathways for the development of breeding programs in the genus *Asparagus*.

## Conclusion

In this study, the mitochondrial genome sequence of garden asparagus was systematically investigated. After the assembling and annotating of its genome, we explored their basic composition, comparative analysis of coding gene differences, codon preference, repetitive sequences and SSRs, gene gain and loss, and sequence diversity analysis. Using the conserved protein-coding genes sequences, its phylogenetic status and evolutionary relationship of garden asparagus were discussed, which provided data support for the taxonomic study and the subsequent innovation and application of germplasm resources in garden asparagus.

## Data availability statement

The data presented in the study are deposited in the NCBI repository, accession number NC_053642.1. This data can be found here: https://www.ncbi.nlm.nih.gov/nuccore/NC_053642.1/.

## Ethics statement

The collection of specimen conformed to the requirement of International ethics, which did not cause damage to the local environment. The process and purpose of this experimental research were in line with the rules and regulations of our institute.

## Author contributions

WS worked on genome assembly, performed the data analysis and wrote the original manuscript. QK designed the project and analyzed the data. WS, JD and CW contributed to the plant sample collection. JD, CW and QK wrote and improved the manuscript. All authors contributed to the article and approved the submitted version.

## Supplementary material

Supplementary Sheet 1 SSR type and number distribution in mitochondrial genome of garden asparagus.

Supplementary Sheet 2 Non-tandem repeat type and number distribution in mitochondrial genome of garden asparagus.

Supplementary Table 1 Plant species used in phylogenetic studies.

Supplementary Table 2 Fragments transferred from chloroplast to mitochondrial genome in garden asparagus.

Supplementary Table 3 Organization of mitochondrial genomes in garden asparagus and other six plants.
