# Supplementary material for: The garden asparagus (Asparagus officinalis L.) mitochondrial genome revealed rich sequence variation throughout whole sequencing data
Source: Front Plant Sci. 2023 Mar 27;14:1140043. doi: 10.3389/fpls.2023.1140043 (PMC10084930; doi:10.3389/fpls.2023.1140043)
Supplement: Supplementary Table 1 — Plant species used in phylogenetic studies. [file Table_1.doc]

**Table S1 Plant species used in phylogenetic studies.**

| Type | Species | Family | NCBI accession number |
| --- | --- | --- | --- |
| Rosids | *Glycine max* | Leguminosae | CM021415.1 |
|  | *Vigna angularis* | Leguminosae | NC_021092.1 |
|  | *Millettia pinnata* | Leguminosae | NC_016742.1 |
|  | *Lotus japonicus* | Leguminosae | NC_016743.2 |
|  | *Medicago truncatula* | Leguminosae | NC_029641.1 |
|  | *Vitis vinifera* | Vitaceae | NC_012119.1 |
|  | *Malus domestica* | Rosaceae | NC_018554.1 |
|  | *Gossypium hirsutum* | Malvaceae | NC_027406.1 |
|  | *Gossypium raimondii* | Malvaceae | NC_029998.1 |
|  | *Carica papaya* | Caricaceae | NC_012116.1 |
|  | *Batis maritima* | Bataceae | NC_024429.1 |
|  | *Brassica napus* | Brassicaceae | NC_008285.1 |
|  | *Arabidopsis thaliana* | Brassicaceae | NC_037304 |
|  | *Raphanus sativus* | Brassicaceae | NC_018551.1 |
|  | *Populus tremula* | Salicaceae | NC_028096.1 |
|  | *Salix suchowensis* | Salicaceae | NC_029317.1 |
|  | *Citrullus lanatus* | Cucurbitaceae | NC_014043.1 |
|  | *Cucumis sativus* | Cucurbitaceae | NC_016005.1 |
|  | *Cucurbita pepo* | Cucurbitaceae | NC_014050.1 |
| Asterids | *Daucus carota* | [Apiaceae](http://www.iplant.cn/info/Apiaceae) | NC_017855.1 |
|  | *Capsicum annuum* | [Solanaceae](http://www.iplant.cn/info/Solanaceae) | NC_024624.1 |
|  | *Nicotiana tabacum* | [Solanaceae](http://www.iplant.cn/info/Solanaceae) | NC_006581.1 |
|  | *Beta vulgaris* | [Amaranthaceae](http://www.iplant.cn/info/Amaranthaceae) | NC_002511.2 |
|  | *Ajuga reptans* | Lamiaceae | NC_023103.1 |
|  | *Salvia miltiorrhiza* | Lamiaceae | NC_023209.1 |
|  | *Asclepias syriaca* | [Apocynaceae](http://www.iplant.cn/info/Apocynaceae) | NC_022796.1 |
| Monocots | *Aegilops speltoides* | Poaceae | NC_022666.1 |
|  | *Allium cepa* | [Amaryllidaceae](http://www.iplant.cn/info/Amaryllidaceae) | NC_030100.1 |
|  | *Butomus umbellatus* | [Butomaceae](http://www.iplant.cn/info/Butomaceae) | NC_021399.1 |
|  | *Cocos nucifera* | [Arecaceae](http://www.iplant.cn/info/Arecaceae) | NC_031696.1 |
|  | *Oryza sativa* | Poaceae | CP018169.1 |
|  | *Phoenix dactylifera* | [Arecaceae](http://www.iplant.cn/info/Arecaceae) | NC_016740.1 |
|  | *Saccharum officinarum* | Poaceae | NC_031164.1 |
|  | *Sorghum bicolor* | Poaceae | NC_008360.1 |
|  | *Spirodela polyrhiza* | Araceae | NC_017840.1 |
|  | *Stratiotes aloides* | [Hydrocharitaceae](http://www.iplant.cn/info/Hydrocharitaceae) | NC_035317.1 |
|  | *Triticum aestivum* | Poaceae | NC_036024.1 |
|  | *Zea mays* | Poaceae | CM025451.1 |
|  | *Zostera marina* | Zosteraceae | NC_035345.1 |
| Ginkgoaceae | *Ginkgo biloba* | [Ginkgoaceae](http://www.iplant.cn/info/Ginkgoaceae) | NC_027976.1 |
| Bryophyta | *Marchantia paleacea* | [Marchantiaceae](http://www.iplant.cn/info/Marchantiaceae) | NC_001660 |
