# Supplementary material for: The garden asparagus (Asparagus officinalis L.) mitochondrial genome revealed rich sequence variation throughout whole sequencing data
Source: Front Plant Sci. 2023 Mar 27;14:1140043. doi: 10.3389/fpls.2023.1140043 (PMC10084930; doi:10.3389/fpls.2023.1140043)
Supplement: Supplementary Table 2 — Fragments transferred from chloroplast to mitochondrial genome in garden asparagus. [file Table_2.docx]

**Table S2** Fragments transferred from cp to mt genome in garden asparagus.

|  | Alignment_length | Identity_% | Mismatches | Gap_opens | mt_start | mt_end | cp_start | cp_end | Gene |
| --- | --- | --- | --- | --- | --- | --- | --- | --- | --- |
| 1 | 2,013 | 100 | 0 | 0 | 347,311 | 349,323 | 105,530 | 107,542 | _ |
| 2 | 2,013 | 100 | 0 | 0 | 347,311 | 349,323 | 136,169 | 134,157 | _ |
| 3 | 1,081 | 96.115 | 25 | 9 | 296,734 | 297,810 | 81,772 | 82,839 | _ |
| 4 | 888 | 76.014 | 160 | 39 | 490,027 | 490,885 | 102,372 | 103,235 | _ |
| 5 | 888 | 76.014 | 160 | 39 | 490,027 | 490,885 | 139,327 | 138,464 | _ |
| 6 | 888 | 74.099 | 177 | 39 | 90,741 | 91,599 | 102,372 | 103,235 | _ |
| 7 | 888 | 74.099 | 177 | 39 | 90,741 | 91,599 | 139,327 | 138,464 | _ |
| 8 | 851 | 97.885 | 15 | 2 | 399,616 | 400,463 | 342 | 1192 | _ |
| 9 | 563 | 79.929 | 57 | 29 | 237,123 | 237,647 | 67,829 | 67,285 | _ |
| 10 | 463 | 75.81 | 78 | 21 | 256,566 | 257,010 | 133,558 | 134,004 | _ |
| 11 | 463 | 75.81 | 78 | 21 | 55,141 | 55,585 | 134,004 | 133,558 | _ |
| 12 | 460 | 71.304 | 118 | 13 | 131,615 | 132,067 | 11,045 | 10,593 | *atp1* |
| 13 | 442 | 76.244 | 71 | 21 | 256,587 | 257,010 | 108,120 | 107,695 | _ |
| 14 | 442 | 76.244 | 71 | 21 | 55,141 | 55,564 | 107,695 | 108,120 | _ |
| 15 | 390 | 91.282 | 24 | 10 | 237,704 | 238,089 | 90,986 | 91,369 | _ |
| 16 | 363 | 78.512 | 68 | 9 | 255,941 | 256,300 | 108,775 | 108,420 | _ |
| 17 | 363 | 78.512 | 68 | 9 | 55,851 | 56,210 | 108,420 | 108,775 | _ |
| 18 | 360 | 78.611 | 67 | 9 | 255,941 | 256,297 | 132,924 | 133,276 | _ |
| 19 | 360 | 78.611 | 67 | 9 | 55,854 | 56,210 | 133,276 | 132,924 | _ |
| 20 | 307 | 97.394 | 8 | 0 | 237,783 | 238,089 | 150,636 | 150,330 | _ |
| 21 | 293 | 92.491 | 16 | 3 | 419,440 | 419,727 | 35,380 | 35,089 | _ |
| 22 | 283 | 83.392 | 42 | 5 | 491,270 | 491,549 | 138,437 | 138,157 | _ |
| 23 | 283 | 77.739 | 57 | 6 | 91,984 | 92,262 | 138,437 | 138,157 | _ |
| 24 | 279 | 79.57 | 37 | 10 | 375,388 | 375,654 | 49,923 | 50,193 | _ |
| 25 | 258 | 84.884 | 36 | 3 | 491,270 | 491,525 | 103,262 | 103,518 | _ |
| 26 | 258 | 78.682 | 51 | 4 | 91,984 | 92,238 | 103,262 | 103,518 | _ |
| 27 | 235 | 80.851 | 29 | 13 | 409,422 | 409,646 | 9,794 | 9,566 | _ |
| 28 | 215 | 91.163 | 19 | 0 | 296,451 | 296,665 | 81,461 | 81,675 | _ |
| 29 | 192 | 74.479 | 45 | 4 | 257,858 | 258,048 | 134,453 | 134,641 | *rrn26* |
| 30 | 192 | 74.479 | 45 | 4 | 54,103 | 54,293 | 134,641 | 134,453 | *rrn26* |
| 31 | 164 | 75.61 | 38 | 2 | 257,904 | 258,066 | 107,202 | 107,040 | *rrn26* |
| 32 | 164 | 75.61 | 38 | 2 | 54,085 | 54,247 | 107,040 | 107,202 | *rrn26* |
| 33 | 143 | 90.909 | 13 | 0 | 125,962 | 126,104 | 107,655 | 107,513 | _ |
| 34 | 143 | 90.909 | 13 | 0 | 125,962 | 126,104 | 134,044 | 134,186 | _ |
| 35 | 97 | 93.814 | 4 | 2 | 160,321 | 160,416 | 110,330 | 110,425 | _ |
| 36 | 97 | 85.567 | 14 | 0 | 258,523 | 258,619 | 106,578 | 106,482 | *rrn26* |
| 37 | 97 | 85.567 | 14 | 0 | 258,523 | 258,619 | 135,121 | 135,217 | *rrn26* |
| 38 | 97 | 85.567 | 14 | 0 | 53,532 | 53,628 | 106,482 | 106,578 | *rrn26* |
| 39 | 97 | 85.567 | 14 | 0 | 53,532 | 53,628 | 135,217 | 135,121 | *rrn26* |
| 40 | 89 | 78.652 | 19 | 0 | 131,291 | 131,379 | 11,342 | 11,254 | *atp1* |
| 41 | 87 | 79.31 | 14 | 1 | 84,365 | 84,451 | 3,977 | 3,895 | _ |
| 42 | 85 | 89.412 | 1 | 4 | 341,141 | 341,217 | 105,714 | 105,798 | _ |
| 43 | 85 | 89.412 | 1 | 4 | 341,141 | 341,217 | 135,985 | 135,901 | _ |
| 44 | 81 | 98.765 | 1 | 0 | 160,336 | 160,416 | 131,354 | 131,274 | _ |
| 45 | 81 | 97.531 | 2 | 0 | 464,703 | 464,783 | 156,234 | 156,154 | _ |
| 46 | 81 | 97.531 | 2 | 0 | 464,703 | 464,783 | 85,465 | 85,545 | _ |
| 47 | 77 | 93.506 | 5 | 0 | 115,544 | 115,620 | 53,359 | 53,435 | *trnM-CAU* |
| 48 | 75 | 81.333 | 14 | 0 | 258,951 | 259,025 | 106,300 | 106,226 | *rrn26* |
| 49 | 75 | 81.333 | 11 | 1 | 376,538 | 376,612 | 151,033 | 151,104 | _ |
| 50 | 75 | 81.333 | 11 | 1 | 376,538 | 376,612 | 90,666 | 90,595 | _ |
| 51 | 75 | 81.333 | 14 | 0 | 53,126 | 53,200 | 106,226 | 106,300 | *rrn26* |
| 52 | 69 | 82.609 | 12 | 0 | 258,951 | 259,019 | 135,399 | 135,467 | *rrn26* |
| 53 | 69 | 82.609 | 12 | 0 | 53,132 | 53,200 | 135,467 | 135,399 | *rrn26* |
| 54 | 65 | 84.615 | 7 | 1 | 426,940 | 427,001 | 110,354 | 110,418 | _ |
| 55 | 65 | 84.615 | 7 | 1 | 426,940 | 427,001 | 131,345 | 131,281 | _ |
| 56 | 64 | 85.938 | 7 | 2 | 491,631 | 491,693 | 103,577 | 103,639 | _ |
| 57 | 64 | 84.375 | 8 | 2 | 92,344 | 92,406 | 103,577 | 103,639 | _ |
| 58 | 62 | 96.774 | 1 | 1 | 10,206 | 10,266 | 40,437 | 40,376 | _ |
| 59 | 60 | 88.333 | 7 | 0 | 489,747 | 489,806 | 102,168 | 102,227 | _ |
| 60 | 60 | 88.333 | 7 | 0 | 489,747 | 489,806 | 139,531 | 139,472 | _ |
| 61 | 60 | 88.333 | 7 | 0 | 90,461 | 90,520 | 102,168 | 102,227 | _ |
| 62 | 60 | 88.333 | 7 | 0 | 90,461 | 90,520 | 139,531 | 139,472 | _ |
| 63 | 59 | 84.746 | 5 | 4 | 24,376 | 24,433 | 48,622 | 48,567 | *trnF-GAA* |
| 64 | 59 | 93.22 | 3 | 1 | 277,921 | 277,979 | 24,592 | 24,649 | _ |
| 65 | 57 | 89.474 | 0 | 4 | 205,969 | 206,019 | 108,385 | 108,441 | _ |
| 66 | 57 | 89.474 | 0 | 4 | 205,969 | 206,019 | 133,314 | 133,258 | _ |
| 67 | 54 | 88.889 | 6 | 0 | 375,293 | 375,346 | 49,810 | 49,863 | _ |
| 68 | 51 | 90.196 | 5 | 0 | 491,643 | 491,693 | 138,110 | 138,060 | _ |
| 69 | 51 | 88.235 | 6 | 0 | 92,356 | 92,406 | 138,110 | 138,060 | _ |
| 70 | 41 | 90.244 | 4 | 0 | 201,521 | 201,561 | 34,646 | 34,686 | _ |
| 71 | 37 | 100 | 0 | 0 | 334,228 | 334,264 | 106,589 | 106,625 | _ |
| 72 | 37 | 100 | 0 | 0 | 334,228 | 334,264 | 135,110 | 135,074 | _ |
| Total | 20,240 |  |  |  |  |  |  |  |  |
