# Supplementary material for: The garden asparagus (Asparagus officinalis L.) mitochondrial genome revealed rich sequence variation throughout whole sequencing data
Source: Front Plant Sci. 2023 Mar 27;14:1140043. doi: 10.3389/fpls.2023.1140043 (PMC10084930; doi:10.3389/fpls.2023.1140043)
Supplement: Supplementary Table 3 — Organization of mitochondrial genomes in garden asparagus and other six plants. [file Table_3.docx]

**Table S3** Organization of mt genomes in garden asparagus and other six plants.

| **Gene** | *Chlorella sorokiniana* | *Funaria hygrometrica* | *Ginkgo biloba* | *Triticum aestivum* | *Asparagus officinalis* | *Nicotiana tabacum* | *Arabidopsis thaliana* |
| --- | --- | --- | --- | --- | --- | --- | --- |
| Size (bp) | 52,528 | 109,586 | 346,544 | 430,597 | 452,526 | 367,808 | 492,062 |
| GC(%) | 29.11 | 40.39 | 50.36 | 44.96 | 44.35 | 44.79 | 45.9 |
| *atp1* | + | + | + | + | + | + | + |
| *atp4* | + | + | + | + | + | - | + |
| *atp6* | + | + | + | + | + | + | + |
| *atp8* | + | + | + | + | + | - | + |
| *atp9* | + | + | + | + | + | + | + |
| *ccmB* | - | + | + | + | + | + | + |
| *ccmC* | - | + | + | + | + | + | + |
| *ccmFC* | - | + | + | + | + | + | + |
| *ccmFN* | - | + | + | + | + | + | + |
| *cob* | + | + | + | + | + | + | + |
| *cox1* | + | + | + | + | + | + | + |
| *cox2* | + | + | + | + | + | + | + |
| *cox3* | + | + | + | + | + | + | + |
| *matR* | - | - | + | + | + | - | + |
| *mttB* | - | - | + | + | + | - | + |
| *nad1* | + | + | + | + | + | + | + |
| *nad2* | + | + | + | + | + | + | + |
| *nad3* | + | + | + | + | + | + | + |
| *nad4* | + | + | + | + | + | + | + |
| *nad4L* | + | + | + | + | + | + | + |
| *nad5* | + | + | + | + | + | + | + |
| *nad6* | + | + | + | + | + | + | + |
| *nad7* | + | + | + | + | + | + | + |
| *nad9* | + | + | + | + | + | + | + |
| *rpl10* | - | + | - | - | - | - | - |
| *rpl16* | + | + | + | + | + | + | + |
| *rpl2* | - | + | + | - | + | + | + |
| *rpl5* | + | + | + | + | + | + | + |
| *rpl6* | + | + | - | - | - | - | - |
| *rps1* | - | + | + | + | + | - | - |
| *rps10* | + | - | + | - | - | + | - |
| *rps11* | + | + | + | - | + | - | - |
| *rps12* | + | + | + | + | + | + | + |
| *rps13* | + | + | + | - | + | + | - |
| *rps14* | + | + | + | - | + | + | - |
| *rps19* | - | + | + | pseudo | + | + | - |
| *rps2* | + | + | + | + | + | - | - |
| *rps3* | - | + | + | + | + | + | + |
| *rps4* | - | + | + | + | + | + | + |
| *rps7* | + | + | + | + | + | - | + |
| *rps8* | - | - | - | - | - | - | - |
| *sdh3* | - | + | + | - | - | + | - |
| *sdh4* | - | + | + | - | - | - | - |
| *trnA-UGC* | + | + | - | - | - | - | - |
| *trnC-GCA* | + | + | + | + | + | + | + |
| *trnD-GUC* | + | + | + | + | + | + | + |
| *trnE-UUC* | + | + | + | + | + | + | + |
| *trnF-GAA* | + | + | + | + | + | + | - |
| *trnfM-CAU* | - | + | + | + | - | + | + |
| *trnG-GCC* | + | + | - | - | - | + | + |
| *trnG-UCC* | + | + | + | - | - | - | - |
| *trnH-GUG* | + | + | + | + | + | + | + |
| *trnI-CAU* | - | + | + | - | - | + | + |
| *trnI-GAU* | + | - | - | - | - | - | - |
| *trnI-UAU* | + | - | - | - | - | - | - |
| *trnK-UUU* | + | + | + | + | + | + | + |
| *trnL-CAA* | - | + | - | - | - | - | - |
| *trnL-UAA* | + | + | + | - | - | - | - |
| *trnL-UAG* | + | + | + | - | - | - | - |
| *trnM-CAU* | + | + | + | + | + | + | + |
| *trnN-GUU* | + | - | - | + | + | + | + |
| *trnP-UGG* | + | + | + | + | + | + | + |
| *trnQ-UUG* | + | + | + | + | + | + | + |
| *trnR-ACG* | + | + | - | - | - | - | - |
| *trnR-UCG* | - | - | - | - | - | - | - |
| *trnR-UCU* | + | + | + | - | - | - | - |
| *trnS-GCU* | + | - | + | - | + | + | + |
| *trnS-GGA* | - | - | - | + | - | + | + |
| *trnS-UGA* | + | + | + | - | + | + | + |
| *trnT-GGU* | - | + | - | - | - | - | - |
| *trnT-UGU* | + | - | - | - | - | - | - |
| *trnV-GAC* | - | - | - | - | - | - | - |
| *trnV-UAC* | + | + | - | - | - | - | - |
| *trnW-CCA* | + | + | + | + | + | + | + |
| *trnY-GUA* | + | + | + | + | + | + | + |
